# Elastin-Derived VGVAPG Fragment Decorated Cell-Penetrating Peptide with Improved Gene Delivery Efficacy

**DOI:** 10.3390/pharmaceutics15020670

**Published:** 2023-02-16

**Authors:** Wen-Juan Shen, Duo-Mei Tian, Le Fu, Biao Jin, Yu Liu, Yun-Sheng Xu, Yong-Bin Ye, Xiao-Bo Wang, Xiao-Jun Xu, Chun Tang, Fang-Ping Li, Chun-Fei Wang, Gang Wu, Le-Ping Yan

**Affiliations:** 1 Department of Critical Care Medicine, The Seventh Affiliated Hospital, Sun Yat-sen University, Shenzhen 518107, China; 2 Department of Dermatovenereology, The Seventh Affiliated Hospital, Sun Yat-sen University, Shenzhen 518107, China; 3 Department of Hematology, Zhongshan Hospital Affiliated to Sun Yat-sen University, Zhongshan 528403, China; 4 Department of Hematology, The Seventh Affiliated Hospital, Sun Yat-sen University, Shenzhen 518107, China; 5 Department of Nephrology, Center of Kidney and Urology, The Seventh Affiliated Hospital, Sun Yat-sen University, Shenzhen 518107, China; 6 Department of Endocrinology, The Seventh Affiliated Hospital, Sun Yat-sen University, Shenzhen 518107, China; 7 Endoscopy Center, The Seventh Affiliated Hospital, Sun Yat-sen University, Shenzhen 518107, China; 8 School of Materials Science and Engineering, South China University of Technology, Guangzhou 510641, China; 9 Scientific Research Center, The Seventh Affiliated Hospital, Sun Yat-sen University, Shenzhen 518107, China; 10 Guangdong Provincial Key Laboratory of Digestive Cancer Research, The Seventh Affiliated Hospital, Sun Yat-sen University, Shenzhen 518107, China; 11 Engineering Research Center of Artificial Organs and Materials, Ministry of Education, Jinan University, Guangzhou 510632, China

**Keywords:** cell-penetrating peptide, gene delivery, elastin-derived peptide, elastin-binding protein, immune modulation

## Abstract

Cell-penetrating peptides (CPPs) are attractive non-viral gene delivery vectors due to their high transfection capacity and safety. Previously, we have shown that cell-penetrating peptide RALA can be a promising gene delivery vector for chronic wound regeneration application. In this study, we engineered a novel peptide called RALA-E by introducing elastin-derived VGVAPG fragment into RALA, in order to target the elastin-binding protein on the cell surface and thus improve delivery efficacy of RALA. The transfection efficiency of RALA-E was evaluated by transfecting the HEK-293T and HeLa cell lines cells with RALA-E/pDNA complexes and the flow-cytometry results showed that RALA-E significantly increased the transfection efficiency by nearly 20% in both cell lines compared to RALA. Inhibition of pDNA transfection on HEK-293T cells via chlorpromazine, genistein and mβCD showed that the inhibition extent in transfection efficiency was much less for RALA-E group compared to RALA group. In addition, RALA-E/miR-146a complexes showed up to 90% uptake efficiency in macrophages, and can escape from the endosome and enter the nucleus to inhibit the expression of inflammation genes. Therefore, the developed RALA-E peptide has high potential as a safe and efficient vector for gene therapy application.

## 1. Introduction

Over the past few decades, gene therapy has been prevalent and considered a promising strategy to prevent and treat various diseases such as cancer, infectious diseases, cardiovascular diseases, etc. [1,2,3]. To this end, it is an urgency to develop gene delivery vectors with low cytotoxicity and high transfection efficiency by overcoming several barriers in the eukaryotic cells, including cellular uptake, endosome escape and nuclear transport. Current gene delivery systems mainly consist of viral and non-viral vectors. Viral vectors are highly effective and dominant in gene delivery, accounting for more than 70% of the vectors used in clinical trials [4], while it is subject to safety problems, such as immunogenicity, inability for fast clearance, and high cost of manufacturing. On the other hand, non-viral vectors are advantageous for their safety, flexibility in packaging nucleic acids and ease production [5]. Common non-viral vectors include inorganic nanoparticles, lipids, polymers, peptides, hybrid systems, etc.

Cell-penetrating peptides (CPPs), consisted of around 5–30 amino acids, are known as highly active non-viral delivery vectors. They are degradable, easy to synthesize and modify, and enable simple formation of non-covalent complex by mixing CPP and nucleic acid. CPPs can penetrate cell membranes and convey various bioactive cargo into the cells via energy-dependent or -independent mechanisms [6]. The most common CPP is the HIV-1 Tat peptide, which has been successfully applied for the delivery of drugs, protein and DNA into cells [7]. RALA is a superior gene delivery CPP developed by McCarthy et al. [6]. It was derived from fusogenic peptides, including glutamic acid-rich GALA and lysine-rich KALA. It has been applied as a vector to develop DNA vaccination for cervical cancer [8], as well as a vector for both FKBPL DNA and RNAi delivery [9]. Under a collaboration with McCarthy, Yan et al. developed collagen/GAG scaffolds activated by RALA-siMMP-9 complexes for improved diabetic wound healing application [10]. It has been reported that histidine possesses strong endosomolytic ability and histidine enriched CPP demonstrated enhanced cellular uptake and endosomal escape [11]. While RALA has only one histidine on its backbone. In order to improve the transfection efficiency of RALA, our group designed a series of peptides named HALA by replacing arginine with histidine within RALA. Among these HALA peptides, HALA2 (WEARLARALARALARHLARALAHALHACEA) showed improved pDNA and siRNA transfection efficiency [12].

Development of safe and high-efficient CPPs is crucial for the clinical application of gene therapy. Enhancement of cellular interaction of CPPs by introduction of cell binding sequences (such as GAG binding sequences) had demonstrated superior efficacy in cargo delivery [13,14]. Dixon et al. developed CPPs with glycosaminoglycan-binding domain to enhance cell interaction and thus improve intracellular translocation [13,14]. Therefore, it would be very interesting to explore if the gene delivery efficacy of RALA can be further improved by using this strategy. VGVAPG is a common peptide sequence derived from an extracellular matrix protein called elastin [15]. During degradation *in vivo*, elastin releases bioactive peptide fragments known as elastin-derived peptides (EDPs) or elastokines [16]. These elastokines mainly bind to the elastin receptor complex on the cell surface and subsequently trigger a variety of cellular functions, including cancer progress [16]. As a common elastokine, VGVAPG can specifically bind to a 67-kDa elastin-binding protein which is a central part of the elastin receptor complex [17]. The VGVAPG sequence has been reported to play important role in the proliferation and differentiation of cells [18,19], cytokine production [20], immune cell modulation [21,22] and angiogenesis [23]. Currently, there is no reported study on exploring this sequence for gene delivery.

Therefore, we aimed to explore if the introduction of cell binding sequence VGVAPG onto the C-terminal of RALA can improve its gene delivery efficiency and the effect of the modified peptide (RALA-E) on cellular behavior.

## 2. Materials and Methods

### 2.1. Preparation of Peptide/Nucleic Acid Complexes

RALA and RALA-E peptides (Table 1) with 98% purity were synthesized by Jiangsu Jitai Peptide industry science and technology Co., Ltd. (Yancheng, Jiangsu, China), supplied as a desalted lyophilized powder. The pDNA (pCMV-C-EGFP) with length of 5010 bp was purchased from the Beyotime (D2626, Shanghai, China). The miR-146a with length of 22 bp (Sequence: UGAGAACUGAAUUCCAUGGGUU) was provided by GenePharma (Suzhou, China).

The complexes were prepared at different N:P ratios ranging from 1 to 12 by adding appropriate amount of peptide solution to the pDNA or miR-146a solutions. Then the peptide/pDNA or peptide/miRNA solutions were mixed by using pipette to form the peptide/pDNA or peptide/miRNA complexes. The complexes were incubated at room temperature for 20 min for stabilization. The peptide, pDNA and miR-146a were dissolved in ultrapure water and the complexes were also prepared in ultrapure water. The final concentrations of pDNA and miR-146a in the above prepared complexes solutions were fixed to 100 nmol/L and 800 nmol/L, respectively. These used concentrations were already optimized from previous studies [10,13]. The N:P ratios of peptide to nucleic acid were calculated based on the molar ratio of the positively charged arginine in the peptide to the negatively charged phosphates in the pDNA/miRNA backbone [9,10]. All complexes were used immediately following their preparation unless otherwise stated.

### 2.2. Agarose Gel Retardation Analysis

RALA/pDNA or RALA-E/pDNA complexes at N:P ratios of 1, 2, 4, 6, 9, 12 were prepared using procedure as mentioned above (The final concentrations of pDNA and miR-146a were fixed to 100 nmol/L). The 1% agarose gel was prepared by adding 0.5 g Agarose (Biosharp, Hefei, China) into 50 mL TAE buffer (Biosharp, Hefei, China), followed by heating until boiling in a microwave oven. Afterwards, electrophoresis of the peptide/pDNA complexes was performed by adding the complexes onto the agarose gel containing Gel Red Nucleic Acid Gel Stain (#11918, Accurate Biology, Changsha, China), under 120 V for 30 min and within the TAE running buffer. Afterward, the gel was visualized by using a ChemiDoc Touch (Bio-rad, Hercules, CA, USA).

### 2.3. PAGE Gel Retardation Assay

Peptide/miRNA complexes at N:P ratios of 1–15 were prepared. The PAGE gel was prepared by using components as mentioned in Table 2. Samples and 5 µL 20 bp DNA ladder (Takara, Kyoto, Japan) was added to the gel. The electrophoresis was run within tris-acetate buffer at 80 V for 2 h. Then the gel was stained in the TBE buffer with 2 µL loading dye (Thermo Fisher Scientific, Waltham, MA, USA). Images were taken using a ChemiDoc Touch (Bio-rad, Hercules, CA, USA) after staining the gel in Gel Red Nucleic Acid Gel Stain solution for 1 h.

### 2.4. Particle Size and Zeta Potential Analysis

Peptide/pDNA and peptide/miRNA complexes at N:P ratios of 1, 2, 4, 6, 9, 12 were formulated as described in Section 2.1 and diluted 20 times (50 µL to 1 mL) by ddH_2_O for the measurement of the particle size, Zeta potential by using a Malvern Zetasizer ZS 3000 (Malvern Instruments, Malvern, Worcestershire, UK).

### 2.5. Serum Stability Evaluation

Peptide/pDNA complexes were prepared at N:P ratio of 9. Then, the complexes were incubated for 1, 2, 3, 4, 5 and 6 h at room temperature with or without 10% fetal calf serum (Gibco, Thermo Fisher Scientific, Waltham, MA, USA) [6]. Subsequently, 10% sodium dodecyl sulfate (SDS, Sigma-Aldrich, St. Louis, MO, USA) was added to decomplex the peptide/pDNA complexes. After incubation for 10 min, samples were electrophoresed within a 1% agarose gel containing GelRed with Tris-acetate running buffer, under 120 V for 30 min. Afterwards, the gel was visualized using a ChemiDoc Touch (Bio-rad, Hercules, CA, USA).

### 2.6. Cell Culture

Human embryonic kidney-293T (HEK-293T), HeLa and THP-1 cell lines were provided by Stem Cell Bank, Chinese Academy of Sciences (Shanghai, China). Real Time-PCR was utilized to detect mycoplasma of these cell lines cells and all the cells used in this study were proofed to be free of mycoplasma. HEK-293T and HeLa cells were cultured in high glucose Dulbecco’s Modified Eagle’s Medium (DMEM, Thermo Fisher Scientific, Waltham, MA, USA) supplemented with 1% penicillin/streptomycin and 10% fetal bovine serum (Gibco, Thermo Fisher Scientific, Waltham, MA, USA) and maintained in an incubator under standard cell culture conditions (37 °C, 90% humidity and 5% CO_2_). THP-1 cells were cultured in Roswell Park Memorial Institute (RPMI) 1640 (Gibco, Thermo Fisher Scientific, Waltham, MA, USA) with 1% penicillin/streptomycin and 10% fetal bovine serum (Gibco, Thermo Fisher Scientific, Waltham, MA, USA) in standard culture conditions.

### 2.7. Macrophage Polarization

THP-1 cells were seeded in 6-well plate with the density of 80% in the RPMI 1640 complete medium and stimulated by Phorbol-12-myristate-13-acetate (PMA, 100 ng/mL; Sigma-Aldrich, St. Louis, MO, USA) for 24 h to differentiation into M0 macrophages. Then the M0 macrophages were polarized toward M1 macrophages by treatment with LPS (1000 ng/mL; Sigma-Aldrich, St. Louis, MO, USA) and IFN-γ (20 ng/mL; Sigma-Aldrich, St. Louis, MO, USA) for 48 h. M1 macrophages were then used for the following transfection essay.

### 2.8. Transfection and Endocytosis Mechanism Studies

For pDNA transfection, 50,000 HEK-293T cells and HeLa cells were seeded per well in a 24-well plate in complete high glucose DMEM approximately 24 h prior to transfection. The media in the wells were removed and replaced by OptiMEM Reduced Serum Medium (GibcoTM, Thermo Fisher Scientific, Waltham, MA, USA) 1 h prior to transfection. Peptide/pDNA complexes were formulated at a N:P ratio of 9 and incubated for 20 min before adding to the cells (final concentration of the pDNA was fixed to 10 nmol/L). Complexes were then incubated with the cells in OptiMEM Reduced Serum Medium for 6 h, before the transfection media were removed and replaced with fresh high glucose DMEM media. Cells were cultured for 2 days before further assessment.

For endocytosis mechanism study, transfection was conducted on HEK-293T cells, under 4 °C or with the addition of either 5 mg/mL Methyl-β-cyclodextrin (MβCD, Aladdin, Shanghai, China), 10 mg/mL Chlorpromazine(Aladdin, Shanghai, China) or 10 mg/mL Genistein (Aladdin, Shanghai, China). Cells after transfection with the addition of various inhibitors or incubated under 4 °C were analyzed by flow cytometer (Beckman Coulter, Brea, CA, USA). Decreasing proportion ratio was calculated according to the following equations (RT: room temperature):

(1) $$\;\mathrm{Decrease}\;\mathrm{of}\;\mathrm{transfected}\;\mathrm{cells}\left(\%\right)=\frac{\mathrm{Transfection}\;\mathrm{efficiency}\left(\mathrm{RT}\right)-\mathrm{Transfection}\;\mathrm{efficiency}\left(\mathrm{with}\;\mathrm{inhibitor}\right)}{\mathrm{Transfection}\;\mathrm{efficiency}\left(\mathrm{RT}\right)}\times 100$$

miRNA transfection was performed on M1 macrophages. The peptide/miRNA complexes were prepared at N:P ratios of 9. The media in the wells were removed and replaced with OptiMEM Reduced Serum Medium (Thermo Fisher Scientific, Waltham, MA, USA) 1 h prior to transfection. M1 macrophage were transfected with the peptide/miRNA complex for 6 h, then the culture medium was changed to RMPI 1640. Non-target miRNA was delivered as a negative control.

### 2.9. Fluorescence Imaging

Fluorescence imaging was conducted by using a Leica DMIL microscope (Leica Microsystems, Wetzlar, Germany) equipped with FITC filter with the magnification of 40 times the actual size. Images of the transfected HEK-293T cells and HeLa cells were taken 2 days after the transfection of the GFP protein-expression pDNA, and images of the M1 macrophages were taken 6 h after transfection of miRNA with FAM- fluorophore.

### 2.10. Flow Cytometry Analysis

For the transfection efficiency experiment, HEK-293T and HeLa cells were firstly detached by using trypsin (Sigma-Aldrich, St. Louis, MO, USA), and then the detached cells were collected by centrifuge (2000 rpm, 3 min) in FACS tubes 48 h after transfection of pDNA. The macrophages were detached by using accutase (Sigma-Aldrich, St. Louis, MO, USA) 6 h after the transfection of miRNA. The detached cells were then washed with FACS Buffer (1× PBS, 0.5% BSA, 2 mM EDTA) by centrifuging at 3500 rpm for 5 minutes under 4  °C. After discarding the supernatant, the cells in each tube were resuspended in 300 μL of FACS buffer. Transfection efficiency was quantitatively assessed by using a flow cytometry (Beckman Coulter, Brea, CA, USA). Non-transfected cells were used as control.

For cytotoxicity test, the cells were washed with FACS Buffer, then resuspended in 100 μL of FACS buffer and dyed by using 1 µL Ghost Dye™ Violet 510 (GV510, Tonbo Biosciences, San Diego, CA, USA) for 15 min in dark on ice. Afterwards, the cells were washed again and resuspended in 300 μL of FACS buffer. Cytotoxicity was detected by using CytoFLEX flow cytometry (Beckman Coulter, Brea, CA, USA).Non-transfected cells were used as control. Data were analyzed by using the FlowJo V10 software (BD Life Sciences–FlowJo, Ashland, OR, USA).

### 2.11. Confocal Microscope Observation

M1 macrophages cells were stained and observed by confocal microscope, after transfection by peptide/miR-146a at N:P 9 for 6 and 24 h. Briefly, the transfected M1 macrophages were washed by PBS (Gibco, Thermo Fisher Scientific, Waltham, MA, USA), then fixed for 10 min at room temperature by using 4% paraformaldehyde solution (Biosharp, Hefei, China). Afterwards, the cells were permeabilized with 0.5% Triton X-100 (Biofroxx, Hesse, Germany) solution for 5 min. In the following, the cells were stained in FITC–conjugated phalloidin solution (diluted 100× in PBS; YEASEN, 40735ES75, Shanghai, China) for 30 min, in dark and under room temperature. After being washed by PBS, cells were incubated in DAPI (1:1000, 1 min, room temperature; Invitrogen, Carlsbad, CA, USA). The stained cells were observed by confocal fluorescence microscopy (LSM 710 NLO, Zeiss, Oberkochen, Germany).

### 2.12. Quantitative Real-Time PCR Assay

Total RNA was isolated from M1 macrophages by using TRIzol^TM^ reagents (Invitrogen, Carlsbad, CA, USA) 36 h after transfection. The reverse transcription of first-strand cDNA was performed as following steps: 37 °C for 15 min, 85 °C for 5 s, and then 37 °C for 10 min by using a PrimeScript^®^RT reagent kit (Takara, Kyoto, Japan) according to the manufacturer’s protocol. Quantitative real-time PCR analysis for mRNA expression of TRAF6, TNF-α, IL-1β and iNOS on macrophages were performed by using SYBR Green PCT Master Mix (Takara, Kyoto, Japan). The mRNA level of β-actin was used as an internal control. The sequences of oligonucleotides used are described in Table 3.

### 2.13. Statistical Analysis

Statistical analysis was performed using Prism 5.0 (GraphPad Software, Inc., 630 La Jolla, CA, USA). Statistically significant differences of data were calculated using the one-tailed unpaired *t*-test with a *p*-value ≤ 0.05 considered as significant. Three independent experiments were performed for each analysis and all the results were presented as mean ± SD (*n* = 3), unless otherwise specified.

## 3. Results

### 3.1. Characterization of the RALA-E/pDNA Complexes

**Figure 1:**
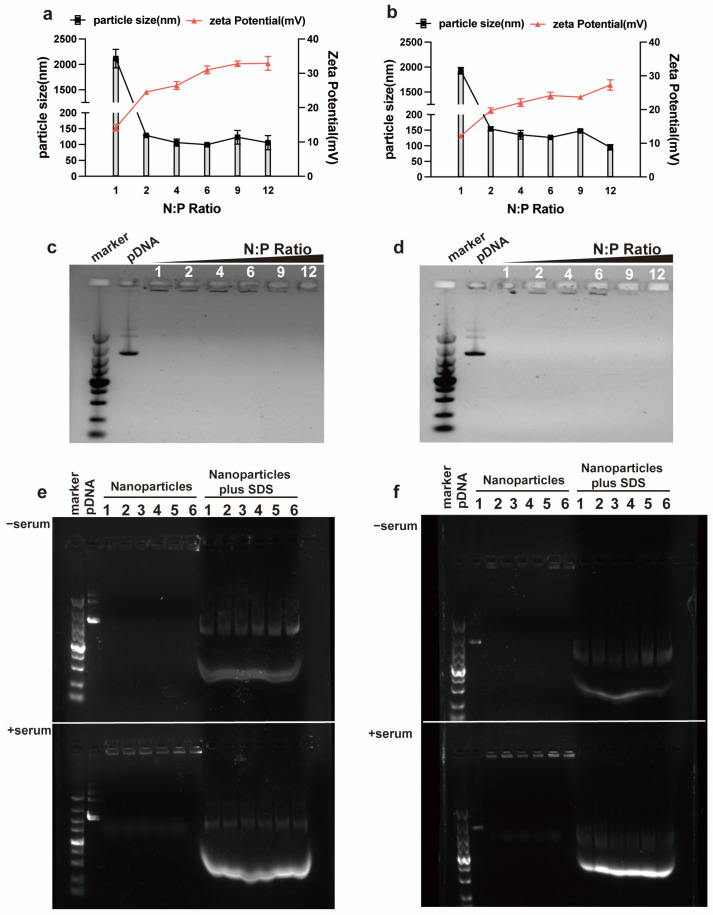
Characterization of RALA-E/pDNA complexes. (**a**,**b**) Particle size and Zeta potential of RALA/pDNA (**a**) and RALA-E/pDNA (**b**) complexes; (**c**,**d**) Gel retardation assay of RALA/pDNA (**c**) and RALA-E/pDNA (**d**) complexes; (**e**,**f**) Serum stability assay of RALA/pDNA (**e**) and RALA-E/pDNA (**f**) complexes. The numbers (1, 2, 4, 6, 9, 12, or 1, 2, 3, 4, 5, 6) in this figure denote N:P ratios.

RALA-E and RALA peptides contain positively charged arginine, while pDNA possesses large amount of negatively charged phosphate. Therefore, the mixing of RALA-E or RALA and pDNA solutions can lead to the formation of complexes through the electrostatic interaction between the positive and negative groups within the peptide and pDNA. As shown in Figure 1a,b, both RALA-E and RALA groups can form complexes of particle size less than 150 nm when N:P ratios was above 2. There were not much differences on the particle size of the complexes within the RALA-E group when the N:P ratios ranged from 2 to 9. Within this N:P ratio range, the particle sizes of RALA-E group were slightly bigger than those of the RALA group at each N:P ratio. The Zeta potential of the RALA-E and RALA groupS increased gradually from around 20 to 27 mV and around 25 to 33 mV as the N:P ratio increased from 2 to 12, respectively.

To investigate the binding stability of the RALA-E/pDNA complexes, nucleic acid gel electrophoresis was performed. From Figure 1c,d, it was found that no decoupled pDNA was observed for both groups in the gel lane at N:P ratios from 1 to 12. These complexes were still stable after incubation in 10% serum for 6 h (Figure 1e,f).

### 3.2. Transfection Efficiency of RALA-E/pDNA in HEK-293T and HeLa Cell Lines

**Figure 2:**
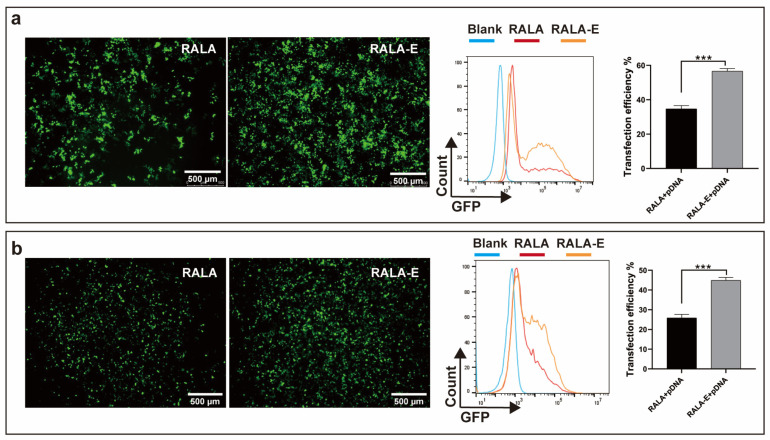
Transfection efficiency of RALA-E/pDNA complexes in 293T and HeLa cell lines. Fluorescent microscope images, flow cytometry histograms and quantitative results of 293T (**a**) and HeLa (**b**) cell lines, 48 h post transfection with RALA/pDNA and RALA-E/pDNA complexes at N:P ratio of 9 (pDNA concentration: 10 nmol/L). *** *p* ≤ 0.001.

To evaluate the transfection efficiency of RALA-E/pDNA, GFP-tagged pDNA was used. The fluorescence areas and intensity were observed by using fluorescence microscope 48 h after transfection, then the proportion of positive cells in HEK-293T cell were quantified by using flow cytometry. Figure 2a showed that there were more green-stained cells in the RALA-E group compared with the RALA group. The flow cytometry results indicated that the transfection efficiency of RALA-E group was close to 60%, while the one of RALA group was less than 40%.

To further verify the superior transfection performance of RALA-E, transfection experiment was also performed on HeLa cell line. As shown in Figure 2b, the transfection efficiency of RALA-E group was close to 45%, while RALA group was only about 25%.

### 3.3. Endocytosis Mechanism of the RALA-E/pDNA Complexes

**Figure 3:**
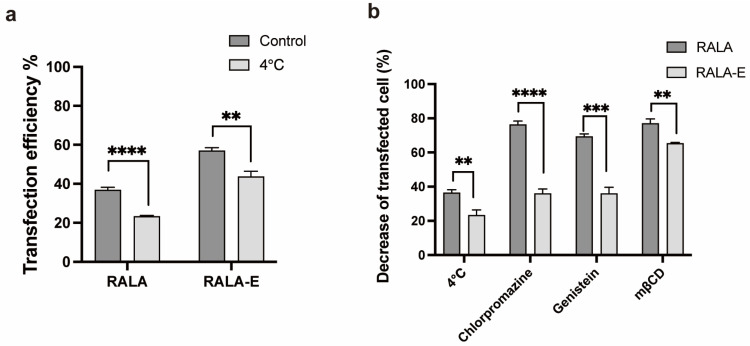
Endocytosis mechanism evaluation of RALA-E/pDNA on HEK-293T cells. (**a**) Transfection efficiency of RALA-E/pDNA under 4 °C; (**b**) Effect of various endocytosis inhibitors on transfection efficiency of RALA-E/pDNA. ** *p* ≤ 0.01, *** *p* ≤ 0.001, **** *p* ≤ 0.0001.

A couple of endocytosis inhibitors for inhibition of different pathways were used to investigate the endocytosis mechanism of RALA-E/pDNA complexes, by using the HEK-293T cells as model cell. In Figure 3a, it was observed that the transfection efficiency of both RALA and RALA-E groups decreased significantly under 4 °C, but the decrease extent was much less in RALA-E group compared to RALA group. In Figure 3b, cell transfection efficiency was reduced for both RALA and RALA-E groups when pre-treating with the three endocytosis inhibitors, but none of the inhibitors can completely inhibit the cellular uptake. In general, RALA group was inhibited by a great extent compared with RALA-E group after inhibition by chlorpromazine, genistein and mβCD. mβCD produced a stronger inhibition effect than the other inhibitors on RALA-E group.

### 3.4. Characterization of the RALA-E/miR-146a Complexes

The above studies investigated the transfection capability of RALA-E/pDNA in HEK-293T and HeLa cell lines by using empty plasmids. Following this, we aimed to explore the delivery ability of RALA-E for specific miRNA, which has been regarded as a small-molecule modulator in gene therapy. To address this, THP-1 derived macrophages and miR-146a were used in this experiment. miR-146a can bind to the TRAF6 mRNA and decrease its expression level.

**Figure 4:**
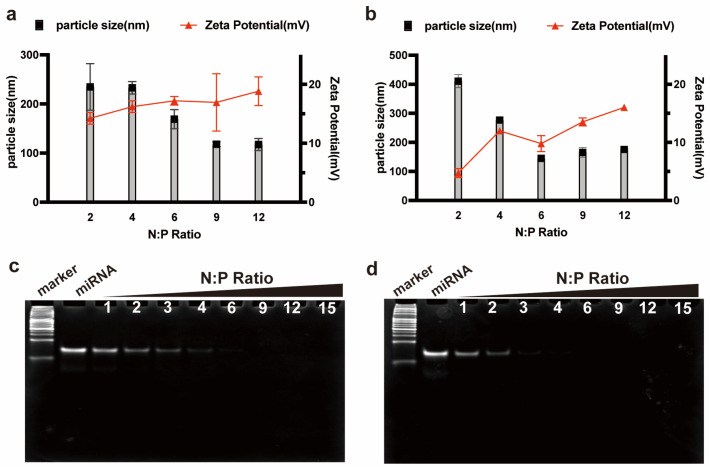
Characterization of RALA-E/miR146a complexes. Particle size and Zeta potential of RALA/miR146a complexes (**a**) and RALA-E/miR146a complexes (**b**). Page gel retardation assay of RALA/miR146a complexes (**c**) and RALA-E/miR146a complexes (**d**). The numbers (2, 4, 6, 9, 12, or 1, 2, 3, 4, 6, 9, 12, 15) in this Figure denote N:P ratios.

The Zeta potential and particle size of peptide/miR-146a complexes were tested first. In Figure 4a,b, it was found that the size of RALA/miR146a complexes decreased from around 230 nm at N:P 2 and 4, to approximately 170 nm at N:P 6, and then to about 110 nm at N:P 9 and 12. While the size of RALA-E/miR146a complexes declined rapidly from 410.6 nm at N:P 2 to 145 nm at N:P 6, then was stabilized at approximately 150 nm. The Zeta potential of RALA/miR-146a complexes slowly increased from 14 mV to 18 mV with the increase of N:P ratio from 2 to 12. In contrast, the Zeta potential of RALA-E/miR-146a complex gradually increased from 5 mV to about 16 mV when N:P ratios increased from 2 to 12. Notably, RALA-E/miR-146a complexes prensented a lower Zeta potential compared with RALA/miR-146a complexes for each tested N:P ratio. Similar trend was also observed in the RALA/pDNA and RALA-E/Pdna complexes. In Figure 1a,b, it was observed that the Zeta potential of RALA-E/pDNA complexes was lower than that of RALA/pDNA complexes at the tested N:P ratios.

As shown in Figure 4c,d, the PAGE gel electrophoresis assay showed that the stability of RALA/miRNA complexes was inferior to the RALA-E/miRNA complexes. When N:P ratio was less than 4, decomplexation of miRNA was observed in the gel lane, indicating a portion of the miR-146a molecules was decoupled. In comparison, no bands were observed in RALA-E/miR146a complexes from N:P 3 upward, illustrating that RALA-E had better combining capacity with miRNA than RALA.

### 3.5. Uptake Efficiency of RALA-E/miR-146a in Macrophages

**Figure 5:**
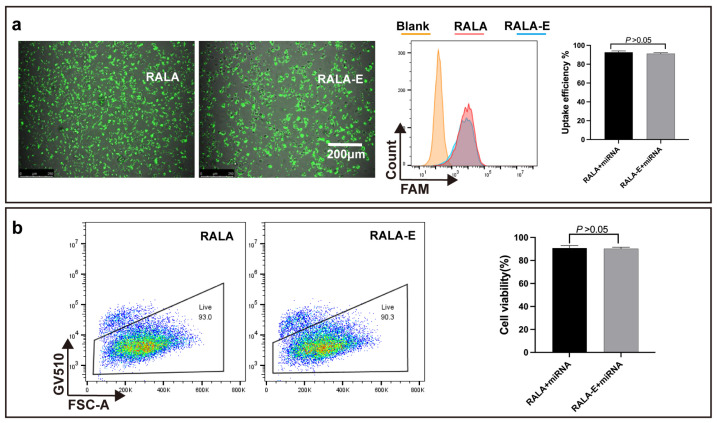
Uptake efficiency and cytocompatibility of RALA-E/miR-146a complex in M1 macrophages. (**a**) Microscope fluorescent image, flow cytometry histograms and quantitative results of M1 macrophages 6 h post transfection with RALA/miR-146a and RALA-E/miR-146a complexes at N:P ratio of 9; (**b**) The proportion of live cells and quantitative results 24 h post-transfection by RALA/miR-146a and RALA-E/miR-146a complexes.

To observe the cellular uptake efficiency of RALA-E/miR-146a complexes, M1 phenotype macrophage was transfected by RALA-E/miR-146a complexes labelled with FAM fluorophore. The transfected cells were observed 6 h after transection. As shown in Figure 5a, bright fluorescence can be observed in both RALA and RALA-E groups. Flow cytometry results showed that the fluorescence intensity of all transfected cells was higher than non-transfected cells. The statistical results denoted that the uptake efficiency of the two groups was about 90%.

In order to verify the cytocompatibility of RALA-E/miR-146a, the transfected cells were stained with GV510 which could stain the dead cells. As shown in Figure 5b. The cell activities of RALA/miR-146a and RALA-E/miR-146a groups were 93% and 90%, respectively, showing good cytocompatibility of these complexes. Additionally, there were no statistical differences between the two groups.

### 3.6. The Dynamic Uptake Process of RALA-E/miR-146a in Macrophages

**Figure 6:**
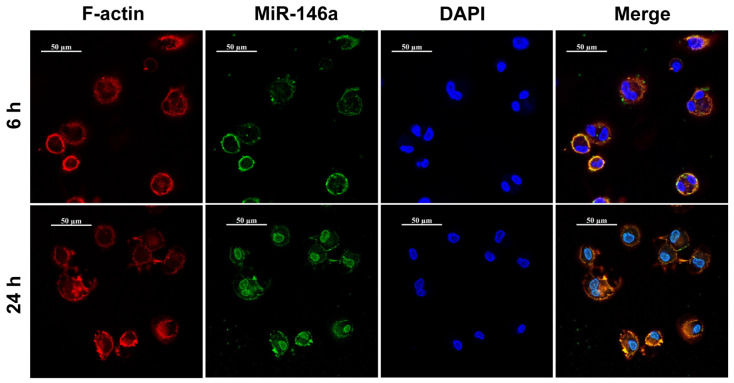
Dynamic uptake process of RALA-E/miR-146a in M1 macrophage 6 and 24 h after transfection.

To observe the dynamic uptake process of miR-146a in macrophages, the cytoskeleton of macrophage was stained with rhodamine labeled phalloidin, and the nucleus of macropahge was stained with DAPI. As shown in Figure 6, the RALA-E/miR-146a complexes mainly existed on the cell membrane 6 h after transfection, and then entered the nuclei 24 h after transfection.

### 3.7. RALA-E/miRNA-146a Can Inhibit the Expression of Targeted Gene Expression

**Figure 7:**
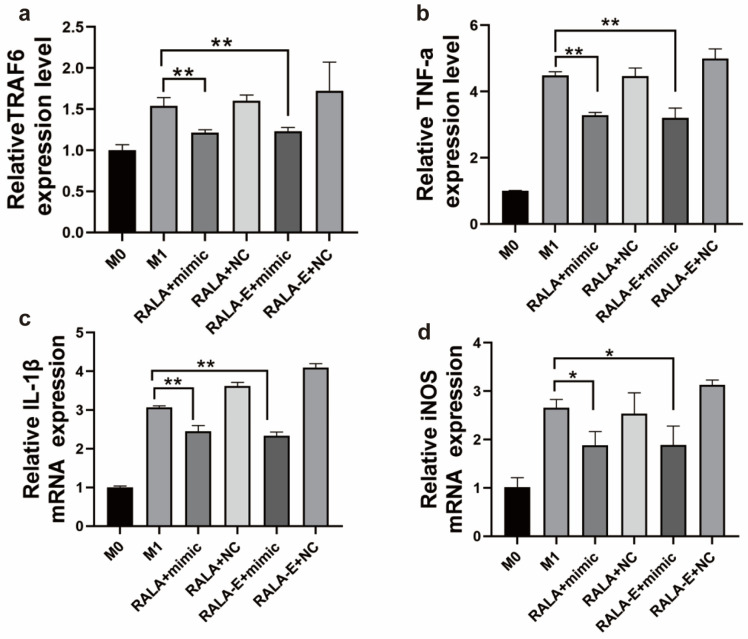
Relative mRNA expression of (**a**) TRAF6, (**b**) TNF-α, (**c**) IL-1β, (**d**) iNOS of M1 macrophages 24 h post-transfection by RALA-E/miR-146a. N = 3. ** *p* ≤ 0.01. * *p* ≤ 0.05.

In order to detect whether RALA-E/miR-146a can play its function after entering the macrophages, the relative mRNA expression levels of TRAF6, TNF-α, IL-1β and iNOS were analyzed by quantitative real-time PCR. In Figure 7, it was fouund that compared with M0 macrophages, the mRNA expression levels of all inflammatory cytokines were significantly increased in M1 macrophage. After treafection by peptide/miRNA146-a complexes, the mRNA expression of these inflammatory cytokines in M1 macrophages were obviously decreased, and no significant differences were observed between the two groups.

## 4. Discussion

In this study, we modified RALA peptide with VGVAPG fragment and generated RALA-E, in order to improve cellular interaction of the CPP/nucleic acid complexes and facilitate cell transfection. Our results showed that such modification significantly increased the transfection efficiency of pDNA in HEK-293T and HeLa cells. Additionally, both RALA and RALA-E peptides can deliver miRNA into macrophages to modulate the expressions of target genes, with good cytocompatibility. Our research illustrates for the first time that modification of CPP with VGVAPG fragment can be a promising strategy for the design of novel CPPs with improved gene transfection efficacy.

Necessary premise for CPPs as gene delivery carrier is their ability to complex the nucleic acid to form stable complexes and maintain the integrity of loaded genes. Our study showed that RALA-E had strong binding capacity to both pDNA and miR-146a. This illustrates that RALA-E is capable to complex nucleic acid of a wide range of length, from less than 30 bp to more than 5000 bp, making it a versatile gene delivery vector.

The particle size and Zeta potential of complexes are key factors affecting cellular uptake. Complexes with the Zeta potential above 10 mV are considered cationic and can produce sufficient interaction with negatively charged cell surface [24], thereby were endocytosed successfully. The weakly positively charged complexes will gather together due to insufficient charge repulsion, which affects the uptake efficiency. RALA contains a high proportion of positively charged arginine, which can bind to negatively charged nucleic acids through non-covalent electrostatic interaction. As the peptide/nucleic acid complexes were prepared at N:P ratio 9 in current study, therefore the complexes possessed a large number of positive charges to facilitate their membrane permeability, making them easy to be taken up. The amino acids within VGVAPG are all neutral, so it has little effect on the charge of the peptide.

Transfection efficiency is one critical aspect to evaluate whether a peptide can be a promising gene delivery vector. Several studies had reported that RALA/nucleic acid complexes possessed excellent transfection efficiency [6,8,10]. In this study, RALA-E significantly improved transfection efficiency of pDNA in HEK-293T and HeLa cell lines compared to RALA, indicating that the VGVAPG modification was effective. Though the VGVAPG modification failed to enhance the uptake ability of miRNA in macrophages, this may be explained by the excellent performance of RALA for small size nucleic acid delivery [10], leaving no much room for further improvement (up to around 90% cellular uptake%). In overall, the high transfection efficiency of RALA-E/nucleic acid complexes supported that RALA-E can be a promising gene delivery vector.

There are two main pathways for the internalization of the CPP/nucleic acid complexes into the cells, namely the endocytic or energy-dependent pathway, and the direct penetration or energy-independent pathways [25]. By performing the transfection under 4 °C, we confirmed that the endocytosis (or energy-dependent pathway) was predominant in cellular internalization process for the complexes in both RALA and RALA-E groups. In the following, we studied the specific endocytosis mechanism of the peptide/pDNA complexes by using various inhibitors.

Clathrin-mediated, caveolae-mediated and lipid raft-mediated endocytosis are main pathways for endocytosis. Chlorpromazine is a common inhibitor for clathrin mediated endocytosis and genistein is able to inhibit caveolae mediated endocytosis [12]. Methyl-β-cyclodextrin (mβCD) can remove cholesterol from the plasma membrane and thus inhibit several pathways, such as cholesterol-enriched microdomains, caveolae and lipid rafts. The less affected transfection efficiency of RALA-E group compared to RALA group after inhibitors treatment, is very likely related with the binding of the VGVAPG sequence to the EBD on the cell surface. The VGVAPG-EBD interaction can act as an anchor for the complex to attach to the cells, which can prolong the staying time of the complexes on the cell surface and thus may greatly facilitate the subsequent endocytosis events. Without this sequence, the attachment of the RALA/pDNA complexes on the cell surface mainly depended on electrostatic interaction and the staying time of the complexes on the cell surface would be much shorter if the complexes were not endocytosed. The mβCD showed superior inhibition effect on RALA-E group compared to other inhibitors. This is probably due to its role on the block of the interaction of VGVAPG sequence and the elastin binding protein. As discussed above, the mβCD can destroy the cholesterol in the plasma membrane. Cholesterol is the key component in the lipid raft. The removal of cholesterol would destroy the microdomains of lipid raft. Rusciani et al. reported that elastin receptor complex and the lipid rafts colocalized on the plasma membrane, and the disruption of or depletion of glycolipids in the microdomain of lipid raft can block the elastin receptor complex signaling [26]. Therefore, the destroy of the lipid raft structure by mβCD would also affect the function or integrity of the elastin receptor complex, and subsequently hindered the binding of VGVAPG sequence on elastin binding protein which is the outer part of the elastin receptor complex.

Successful gene delivery vectors need to be able to effectively overcome the membrane barrier of cells and deliver genes to characteristic organelles to play biological functions within cells. Therefore, a desirable vector should be able to enter the nucleus in a short period of time without being degraded by lysosomal enzymes. Most of the loaded miR-146a can successfully enter the nucleus 24 h after the transfection, indicating that the modified peptide had good endosomal escape ability.

The successful modulate the target gene expression by delivered miR-146a is the ultimate goal of this study. The inhibition results on target genes showed that RALA-E/miR-146a complexes can successfully inhibit the expression of inflammatory factors of M1 macrophages, further proving the potential of RALA-E as a gene delivery vector for clinical application.

In the next step, we plan to incorporate the RALA-E/nucleic acid complexes into elastin-based hydrogels which were recently developed by our group [27], as a biological regenerative and sustained release platform, to regulate the inflammatory microenvironment and improve the healing of chronic wounds.

## 5. Conclusions

In the current work, we developed a novel CPP RALA-E by decorating RALA peptide with elastin-derived VGVAPG fragment. RALA-E showed excellent pDNA and miRNA complexation capacity, desirable gene protection capability and good cytocompatibility. It can efficiently complex pDNA or miRNA to form complexes with suitable particle size and surface charge for cellular uptake. Additionally, VGVAPG modification significantly enhanced cellular internalization via interaction with elastin binding protein and thus promoted pDNA transfection efficiency in HEK-293T and HeLa cell lines. Moreover, the RALA-E/miRNA complexes can be transported into the nuclear of M1 macrophages and significantly inhibit the expression of target genes of macrophages. Therefore, the developed RALA-E can be a promising gene delivery vector for regenerative medicine or diseases healing applications.

## Figures and Tables

**Table 1:** The sequence of RALA and RALA-E peptides.

Peptide	Sequence
RALA	N-WEARLARALA RALARHLARA LARALRACEA-C
RALA-E	N-WEARLARALA RALARHLARA LARALRACEAVGVAPG-C

**Table 2:** PAGE gel constituent.

Acrylamide 30%	10× TBE	ddH2O	TEMED	Ammonium Persulfate 10%
33.33 mL	5 mL	11.67 mL	25 µL	150 µL

**Table 3:** mRNA sequence primers used in the RT-qPCR assays.

Gene	Sequence (Forward)	Sequence (Reverse)
TRAF 6	TTGCTCTTATGGATTGTCCCC	TTTGCTCTTATGGATTGTCCCC
TNF-α	CCTCTCTCTAATCAGCCCTCTG	GAGGACCTGGGAGTAGATGAG
IL-1β	TTCGACACATGGGATAACGAGG	TTTTTGCTGTGAGTCCCGGAG
iNOS	TCATCCGCTATGCTGGCTAC	CCCGAAACCACTCGTATTTGG

## Data Availability

The datasets used within the current study are available from the corresponding author on reasonable request.
